# The Imminent Fall of Home-Office Workers During COVID-19 Outbreak: Suggestions to Cope With Burnout

**DOI:** 10.3389/fpsyg.2022.647418

**Published:** 2022-04-06

**Authors:** Douglas da Silveira Pereira, Fabiana Silva Ribeiro

**Affiliations:** ^1^Faculty of Education, University of São Paulo, São Paulo, Brazil; ^2^Department of Social Sciences, University of Luxembourg, Esch-sur-Alzette, Luxembourg

**Keywords:** COVID-19, home-office, burnout, coping, read, mindfulness, social media

## Introduction

On 11 March 2020, the World Health Organization (WHO) declared the coronavirus 2019 (COVID-19) a pandemic. From then on, governments around the globe started to deal with the impact caused by this disease. The SARS-CoV-2, the virus responsible for the COVID-19, can cause a range of symptoms such as fever, dry cough, fatigue, anosmia, or, in complex cases, severe acute respiratory syndrome (Menni et al., 2020). Furthermore, considering that it is easily transmitted and, therefore, can lead to overcrowding of the health systems (Driggin et al., 2020), many countries had to adopt from simple safety measures to hard ones, for instance, from incentivizing hand sanitation and use of masks for individual protection to social distance, isolation, and lockdown, which quickly became a part of everyday people's lives (World Health Organization, 2021). As a consequence, schools were closed, and companies started asking their employees, when possible, to work from home (Spurk and Straub, 2020). In this context, we can highlight at least two different realities. The first is composed of those who need to keep commuting to work and, therefore, exposing themselves to the virus, either to fight the pandemic as the healthcare professionals or just for maintaining their income, as, for instance, couriers and app drivers (Hossain, 2021). The second group includes those who could start working from home, but in this case, they had to fight against another kind of challenge (Hayes et al., 2020).

Although the vaccination against COVID-19 is being carried out, a study has shown that the willingness to be vaccinated worldwide is estimated at around 66% of the population (Nehal et al., 2021), which means that due to various factors, it will be a difficult task to vaccinate everyone quickly (Chakraborty et al., 2021). For this reason, it is necessary to maintain vigilance on the numbers of COVID-19 cases, which may lead to intermittent social distancing or partial lockdowns in 2022, according to the predictions (Kissler et al., 2020; Liu et al., 2021).

Throughout the pandemic, the healthcare professionals assumed their positions in the frontline, embracing those in need of care. Yet, various studies showed that healthcare professionals presented a higher risk of developing posttraumatic stress disorder, especially those with less experience in their profession (Carmassi et al., 2020; Lai et al., 2020), also anxiety, depression, and insomnia symptoms (Giorgi et al., 2020; Pollock et al., 2020; Preti et al., 2020; Spoorthy et al., 2020). Moreover, Vizheh et al. (2020) argued that although healthcare professionals presented a high prevalence of psychological disorders, most of them were not receiving psychological support or performing activities to improve their mental health.

In addition, in their narrative review, Giorgi et al. (2020) proposed that besides the healthcare and frontline workers, young adults and migrant workers were also vulnerable to mental health problems during COVID-19. The authors suggested strategies that could improve performance and increase wellbeing, that is, smart working, reduction in working hours, the inclusion of safe protocols, and improved support from the leaders.

Although we have more access to the psychological effects of the pandemic on healthcare and frontline professionals, less is known about the impact on other workers (i.e., office workers, self-employed professionals, or educators). Additionally, beyond the fear of unemployment and the economic implications (Kawohl and Nordt, 2020), some other consequences on mental health due to the implementation of home office also have been shown (Hayes et al., 2020), such as the lack of clear boundaries between personal and professional life, which ends up influencing the workload (Liu and Lo, 2018; Irawanto et al., 2021).

For this reason, we would like to discuss some strategies for these home office workers to deal with the psychological issues, that is, burnout, caused by the advent of the COVID-19 outbreak. Furthermore, it is essential to highlight that the strategies posited here were not intended to replace mental healthcare professional accompaniment.

## COVID, Home Office, and Burnout

While people try to find a way to live in this new imposed reality, a silent consequence begins to spread around us. People are feeling more and more exhausted, as shown by the study of Shevlin et al. (2020). Although the home office is a very effective way to reduce and prevent the spread of COVID-19, it is essential to highlight that the professionals do not experience a favorable environment since in a significant part of the cases, concomitantly, workers should also be parents or caregivers during working hours (Eddleston and Mulki, 2017).

Apart from being parents, they have to deal with different tasks such as managing work goals, domestic activities, and their own children's education (Garbe et al., 2020). Moreover, since most schools are closed or closing intermittently, parents also have to work as the private teachers (Garbe et al., 2020). Consequently, exhaustion emerges due to the high demand for responsibilities, such as family and work. In this way, workers tend to move away emotionally from the people involved in these previously cited environments, which is one of the burnout syndrome elements (Mikolajczak et al., 2018). In the end, they question their own skills in the face of unsatisfactory work results (Roskam et al., 2018). Besides, they have to deal with the risk of getting ill, the possibility of losing a loved one, or not being available for their family. Together, all these situations can result in high levels of stress and anxiety (Lindblom, 2006; Hayes et al., 2020), which are highly associated with the development of burnout syndrome (Koutsimani et al., 2019).

The World Health Organization (2019) describes burnout as a syndrome that results from persistent stress at work that has not been effectively managed, which was formally recognized by the medical community last year as an official occupational syndrome. According to Maslach (1993), this psychological syndrome has at least three elements: feelings of exhaustion, negativism or cynicism related to their job, and low performance at work. Although, the number of studies investigating the prevalence of burnout in the general population is scarce, some studies showed that burnout prevalence ranged from 7 to 13.7% (Kalimo, 2000; Kant et al., 2003), having as the risk factors the age and working for more than 40 h a week.

Regarding the psychological effects, burnout can be mistaken for depression (Koutsimani et al., 2019) and associated with lower cognitive performance, commitment, and/or motivation (Kaschka et al., 2011). In addition, burnout has been highlighted in several articles as one of the mental effects of the COVID-19 pandemic in healthcare workers at the frontline (de Pablo et al., 2020). Nevertheless, the researchers have paid less attention to other classes of workers, such as the professionals who are working from home and have been suffering the consequences of social distancing measures potentializing or developing the burnout syndrome (Hayes et al., 2020).

## Suggestions on How to Cope With Burnout

The main interventions promoted to prevent or deal with burnout can be person-directed (group or individual), that is, aiming to enhance job competencies or emotion regulatory skills through cognitive behavioral procedures or organization-directed, that is, restructuring of work demands and organization, or a combination of both (Awa et al., 2010; Dreison et al., 2018). The previous systematic reviews revealed that the most useful intervention applied is person-directed with an individual focus seems to be the most effective in the short term (Awa et al., 2010; Dreison et al., 2018).

However, it is essential to highlight that even before the COVID-19 pandemic, person-directed interventions to prevent or to cope with burnout were scarcely provided by the employees (Hofer et al., 2017). For this reason, individually focused strategies such as reading, mindfulness, and less use of social media could increase workers' resilience preventing worse mental health consequences. These strategies were shown to be effective in preventing and/or decreasing burnout symptoms, according to the scientific literature (Luken and Sammons, 2016; Hunt et al., 2018; Marchalik et al., 2019).

### Reading

Social isolation exposed us to a higher number of demands, worries about health, cleaning, work, family, and daily activities (Brooks et al., 2020). Reading can be an optimal choice when an escape from today's life is necessary (Hofer et al., 2017). Reading books, that is, novels, poetry, biography, fiction, improves concentration, reduces anxiety, and puts the energy in only one element, the history, in front of the reader (Stip et al., 2020).

Reading, especially the novels, might be a protective strategy against burnout. A study performed with healthcare professionals showed that reading non-medical literature on a consistent basis was associated with a significantly decreased likelihood of developing burnout (Marchalik et al., 2019). In addition, one of the results of this study showed a protective effect of the literature across depersonalization. These findings point to the possibility of the literature to nurture empathy and reduce health providers' callousness toward their patients (Marchalik et al., 2019). Reading can help us to keep our minds organized even in the middle of chaos and improve our social skills.

In fact, reading books can also improve life expectancy. If we still cannot confirm the popular saying “one apple a day keeps the doctor away”, scientific literature already indicated the benefits of one chapter a day for the quality of life and longevity (Bavishi et al., 2016). In research done for over 10 years, the reading habit of more than three thousand people showed that reading books has a cognitive protective effect. The immersive nature of reading books would keep our cognitive status, which provides a survival advantage (Bavishi et al., 2016). This study showed that 30 min a day is enough to get this advantage.

### Mindfulness

Mindfulness is the awareness that comes from the attention directed, with a purpose, to our thoughts, without judgment, and being aware of every moment experienced (Kabat-Zinn, 2003). In other words, practicing mindfulness means living the present moment with no judgment, just being able to recognize and acknowledge it. The practice of mindfulness can be developed systematically or unsystematically (Luken and Sammons, 2016).

In fact, substantial evidence of the positive impact of mindfulness in decreasing burnout symptoms has been shown in the previous studies. For example, Luken and Sammons (2016) carried out a systematic review exploring the mindfulness practice on burnout. Though the eight included studies were mainly with workers from the healthcare sector, it evidenced significant reductions in burnout symptoms after mindfulness training. One of the studies selected was carried out with workers from varied sectors, that is, bankers, retail salespersons, etc., without any previous training. The authors applied a self-guided mindfulness training that comprised mindfulness meditation and informal daily exercises for 10 days. As a result, the experimental group reported less emotional exhaustion and higher job satisfaction levels (Hülsheger et al., 2013). Moreover, a study that explores the effects of a brief web-based intervention of mindfulness, carried out for 15 min a day, for 6 days a week during 1 month and half period, found that stress and burnout symptoms decreased in the postintervention assessment (Eriksson et al., 2018).

The authors' main explanation is that those individuals paying attention to the present moment with a non-judgmental attitude might observe the situations objectively, which decreases the levels of bias, negative perceptions, or appraisal of the events. In the context of work, mindfulness individuals can produce a positive appraisal of stressful and challenging circumstances, which might increase job satisfaction (Hülsheger et al., 2013). Another point raised by Shapiro et al. (2006) is the contact that mindfulness establishes with individual values and needs.

### Reduce Social Media

During the COVID-19 outbreak, people started spending more time on social media during social distancing measures (González-Padilla and Tortolero-Blanco, 2020). However, although social media is a way to be connected and decrease the levels of loneliness, as well as to obtain information and education, it is important to point out that despite these positive factors it can create an emotional bond with social media platforms, on the other hand, it could also cause addictive tendencies (Brailovskaia and Margraf, 2021), and it has an essential role in the development of burnout syndrome through social comparison (Vogel et al., 2014).

Furthermore, the excessive use of social media can have additional detrimental effects due to an overload of COVID-19-related information that can be misleading, causing a powerless sense, which can increase mental fatigue (Liu and Ma, 2018; Wiederhold, 2020), leading to emotional problems such as depression and anxiety (Liu and Ma, 2018; Hossain et al., 2020; Ni et al., 2020) and low sleep quality (Garett et al., 2018). It also increases management complexity and workload (Fusi and Feeney, 2018).

The recent literature has shown an advantage in decreasing social media use regarding improving wellbeing, a healthy lifestyle, and reducing depression levels, all characteristics that underlie burnout syndrome. For instance, Hunt et al. (2018) accompanied 143 participants allocated into two groups, those who used Facebook, Instagram, and Snapchat, each for about 10 min a day, and another group without limitation. They revealed that the group with social media limitations showed less loneliness and depression after 3 weeks of decreased social media use. In this context, the authors suggest that the limitation of 30 min a day using social media can substantially improve wellbeing. Another study published in 2020 goes in the same line since it evidences that limiting Facebook use to 20 min a day during at least 3 months can improve life satisfaction, frequency of physical activity, and less depressive symptoms, which lead the authors to conclude that the reduced access to social media might improve wellbeing and healthy behaviors (Brailovskaia et al., 2020).

As previously cited, burnout has been linked to the low levels of wellbeing (Levi, 1995; Cotton, 2003) and high symptoms of depression (Koutsimani et al., 2019). Therefore, we propose that 20–30 min of less access to social media can help diminish the impact of burnout in home-office workers during pandemics.

## Conclusion

In this opinion article, we propose three different strategies to promote emotional relief, appraisal, decrease of distraction, and anxiety levels. In conclusion, we would like to emphasize that it is a hard time for everyone to live in a pandemic situation, and it is necessary to find strategies to survive this period with the best possible mental health. As human beings, the way we use our time defines us, as well as our health. For this reason, we recommend replacing the part of the time spent on social media with fiction readings (such as novels for at least 30 min a day) or doing mindfulness meditation (for 15 min a day). These activities are low cost, well supported by the scientific literature, and can have a very positive impact on those aiming to prevent burnout, reduce or even eliminate burnout symptoms, and contribute to their wellbeing.

## Author Contributions

All authors listed have made a substantial, direct, and intellectual contribution to the work and approved it for publication.

## Conflict of Interest

The authors declare that the research was conducted in the absence of any commercial or financial relationships that could be construed as a potential conflict of interest.

## Publisher's Note

All claims expressed in this article are solely those of the authors and do not necessarily represent those of their affiliated organizations, or those of the publisher, the editors and the reviewers. Any product that may be evaluated in this article, or claim that may be made by its manufacturer, is not guaranteed or endorsed by the publisher.
